# The Last Decade Publications on Diabetic Peripheral Neuropathic Pain: A Bibliometric Analysis

**DOI:** 10.3389/fnmol.2022.854000

**Published:** 2022-04-13

**Authors:** Shu-Hao Du, Yi-Li Zheng, Yong-Hui Zhang, Ming-Wen Wang, Xue-Qiang Wang

**Affiliations:** ^1^Department of Sport Rehabilitation, Shanghai University of Sport, Shanghai, China; ^2^Department of Rehabilitation Medicine, Shanghai Shangti Orthopaedic Hospital, Shanghai, China

**Keywords:** diabetic, neuropathic pain, trend, visual analysis, bibliometric

## Abstract

**Background:**

Diabetic peripheral neuropathic pain (DPNP) is a usual complication of diabetes with a high incidence and mortality. Many diabetes-related studies have been published in various journals. However, bibliometrics and visual analyses in the domain of DPNP research are still lacking. The study aimed to offer a visual method to observe the systematic overview of global research in this field from 2011 to 2021.

**Methods:**

The publications from the Science Citation Index Expanded in Web of Science (WOS) in the past 11 years (from 2011 to 2021) were collected and sorted out, and those related to DPNP were extracted and analyzed. The article language was limited in English. Then, CiteSpace V was used for the bibliometric analysis of the extracted literature.

**Results:**

A total of 1,422 articles met the inclusion criteria. A continuous but unstable growth in the amounts of papers published on DPNP was observed over the last 11 years. The subject sort of the 1,422 papers mainly concentrates on *Endocrinology Metabolism*, *Clinical neurology* and *Neurosciences* from the WOS. According to the research contribution in the field of DPNP, the United States occupies a leading position, with the highest amounts of publications, citations, open access, and the H- index.

**Conclusion:**

This study provides a visual analysis method for the trend of DPNP, and offers some hidden serviceable information that may define new directions for future research.

## Introduction

Diabetes mellitus refers to a series of metabolic diseases distinguished by high blood sugar, which is mainly caused by lack of insulin secretion, defective action, or both (Abbott et al., 2011). According to the reports, diabetes accounted for the largest share among 155 conditions of United States health care spending in 2013, with an estimated amount of $101.4 billion, including 57.6% spent on medical drugs and 23.5% on outpatient care (Dieleman et al., 2016). Diabetic peripheral neuropathic pain (DPNP) is a common diabetic peripheral neuropathy, and about 20–30% of patients with diabetes experience peripheral neuropathic pain during their lifetime (Baba et al., 2019). Moreover, more than half of the diabetic patients may admit peripheral neuropathic pain (Calcutt, 2010). Most studies found that it developed before pre-diabetes (Tesfaye et al., 2010). There is evidence that DPNP reduces health-related quality of life and increases health-care expenditures (Gore et al., 2006; Stewart et al., 2007; Ritzwoller et al., 2009). Furthermore, DPNP is closely associated with some depressive symptoms, anxiety, and lower rates of optimal sleep (Sadosky et al., 2013).

Due to the high morbidity and family burden of DPNP, an increasing number of studies have been involved in the field of DPNP, and relevant literature has been published in academic journals. Some studies have explored the mechanisms of pain relief by non-pharmacological interventions (Zheng et al., 2021; Peng et al., 2022; Wu et al., 2022). However, only a few large-scale systematic global reviews of DPNP have been published (Çakici et al., 2016).

Bibliometric analysis is an important quantitative analysis method for literature on a specific topic (Bornmann and Leydesdorff, 2014). The purpose of this work is to offer a classified analysis of this research on DPNP from 2011 to 2021, including quantitative information by authors, countries, institutions, and co-citations (Chen and Wang, 2020). Bibliometrics analyzes the numbers of articles published, keywords, citations, and cooperation in recent years to better understand the current trends and the knowledge structure of research. WOS is a database from which relevant literature can be extracted, and CiteSpace V is used for the in-depth analysis of visualization (Yao et al., 2020). Moreover, we can know which places occupy the international leading positions in this field so far and which one has a significant impact on this discipline through the analysis of publishing states, authors, and institutions (Chen et al., 2012, 2014; Li et al., 2020). Visual analysis extracts useful information from big data through data mining technology and presents it clearly for readers to understand the development of the discipline more intuitively (Liao et al., 2018; Weng et al., 2020). Bibliometrics and visual analysis have been widely used in various fields, such as mathematics (Sabermahani and Ordokhani, 2021), medicine (Weng et al., 2020), artificial intelligence (Xieling et al., 2021), big data financial decision-making (Nobanee, 2021) and economics (Liang et al., 2017). According to these researches, this study explored the characteristics of articles in the field of DPNP through bibliometrics and visual analysis. Specific influential literature can be found to evaluate the current research status of diabetic neuropathy and predict the future research direction through the association among different literature and citations. The study also provided valuable reference information for researchers and promoted the cooperation among various institutions (Weng et al., 2020).

## Materials and Methods

### Source and Search Strategy

We retrieved and downloaded all published literature in the past 11 years (from 2011 to 2021) from the Science Citation Index Expanded (SCI-Expanded) of WOS. The keywords “pain” and “diabetes” and its disparate utterances were used as the subject to search correlative paper (Weng et al., 2020). All fundamental information about each article, such as authors, countries, institutions, citation, key words, and references, was collected (Weng et al., 2020). The specific search strategy can be found in the Supplementary.

### Inclusion Criteria

The included paper should meet the following standards: (1) Articles published in various journals over the last 11 years (from 2011 to 2021); (2) Articles in English; and (3) literature on pain and diabetic.

### Analysis Tool

Microsoft Office Excel was used to extract the literature downloaded from WOS. The extracted relevant data included the amounts of publications and citations from different countries/regions, and institutions; keywords; journals; references; and H-index. Two documents named “Date” and “Project” were created to extract the data downloaded from the WOS. CiteSpace V, a superb bibliometric analysis instrument, was used for the bibliometric analysis of extracted literature. We diagrammed co-citation diagrams of authors, countries, and institutions that contributed the most to diabetic peripheral neuralgia. The top 25 keywords with the strongest citation bursts and the top 10 citations paper were drawn. The numbers of publications, citations, and H-indexes for different years were also summarized and an annual bar chart was plotted. Meanwhile, a regional world diagram was drawn using Microsoft Office Excel. The red areas represented the regions with the highest amounts of posts, followed by orange, purple, yellow, and blue. The gray area represented a region that had no published literature. Cluster analysis and citation bursts were performed and a large node circle indicated a high occurrence frequency.

## Results

### The Number and Growth Trend of Annual Publications

**FIGURE 1:**
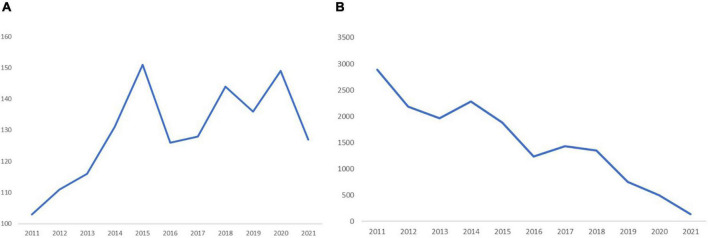
The amounts of papers and citations published. **(A)** The annual amounts of publications on DPNP research from 2011 to 2021. **(B)** The annual amounts of citations on DPNP research from 2011 to 2021.

**FIGURE 2:**
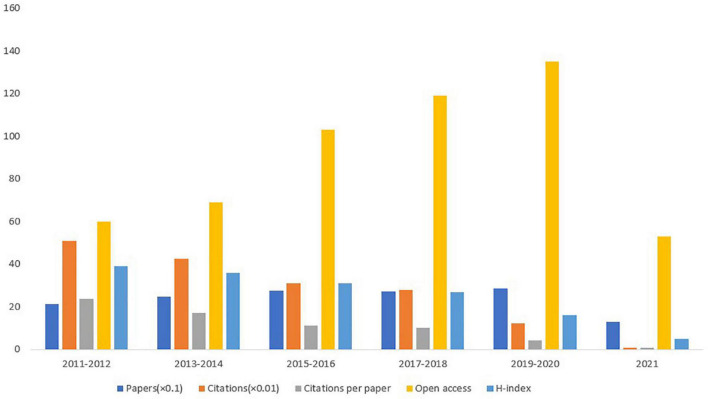
Amounts of publications, citations, citations per paper, open access paper, H-index, and citations for each 2-year time period.

A total of 1,422 articles met the inclusion criteria. We summarized the amounts of publications and citations from 2011 to 2021 and charted the growth trend (Figure 1). The numbers of papers published each year has risen from 103 in 2011 to 127 in 2021 (Figure 1A). Overall, the number of published papers showed a continuous but unstable growth trend, which can be roughly divided into two stages. In the first stage, the number of published papers increased steadily from 103 in 2015 to 151 in2015. From 2015 to 2016, with the number of published papers dropping from 151 to 126, was a turning point. The period from 2016 to 2021 was the second stage, showing a fluctuating growth and a relatively slow growth rate, with an average annual publication volume of 135. The 1,422 papers were cited 16.620 times. The numbers of citations have decrease from 2,893 in 2011 to 138 in 2021 (Figure 1B). Considering the impact of publication year, the absence of high amounts of citations in recent years was normal. In the last five 2-year periods (2011–2012, 2013–2014, 2015–2016, 2017–2018, 2019–2020, and 2021), the largest average numbers of citations per paper (23.74), citations (5,080), and the highest H-index (39) occurred in 2011–2012. In 2019–2020, the highest numbers of published papers and open access values were 285 and 135, respectively (Figure 2). The amounts of papers published and open access in the domain of DPNP increased continuously but unstably, while the citation per paper and H-index decreased.

### Different Subject Sort of Web of Science

**FIGURE 3:**
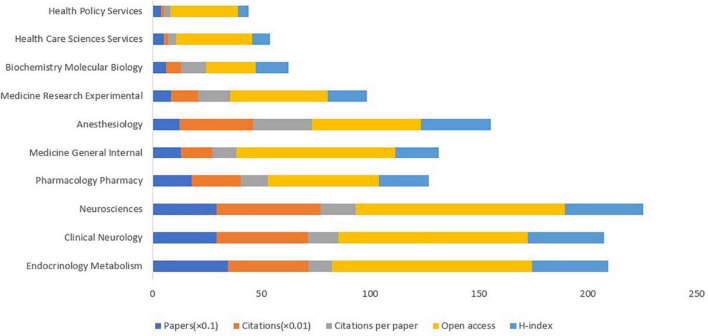
The amounts of papers, citations, citations per paper, open access papers, and H-index of the top 10 subject sort of Web of Science.

The top 10 subject sorts on DPNP, including publications, citations, open access, and H-index, were demonstrated in Figure 3. Among the top 10 discipline sorts, *Endocrinology Metabolism* published most papers, with amount of 347. However, *Neurosciences* had the highest amounts of citations, the open access papers, and the H-index, being 4,771, 96, and 36, respectively. *Anesthesiology* had the highest amounts of citations per paper of 27.22. According to statistical analysis, the top 10 subject sorts calculated by the amounts of publications were *Endocrinology Metabolism, Clinical neurology, Neurosciences, Pharmacology pharmacy, Medicine general internal, Anesthesiology, Medicine research experimental, Biochemistry molecular biology, Health care sciences services*, and *Health policy services.*

### Distribution by Different Journals

The Table 1 demonstrated the top 10 journals in the field of DPNP. In the top 10 journals, *Diabetes*, *Diabetologia*, and *Diabetic medicine* contributed the most to the amounts of published papers, with the papers of 65, 59, and 42 respectively. *Pain* showed the most citations and the highest H-index, being 1,638 and 19, respectively. *Diabetes care* had the largest average per paper citations and the highest impact factor, which were 66.53 and 19.112, respectively. *Value in health* presented the highest open access value of 27 to the public free of charge. Among the top 10 journals, 45.45% were Q1 (Q1 on behalf of the top 25% of 3-year average IF of various journals), 27.27% journals were Q2 (Q2 on behalf of the 25–50% of average IF distribution), and 27.27% journals were Q3 (Q3 on behalf of the 50–75% of average IF distribution).

**TABLE 1:** The top 10 journals in the field of DPNP.

Journals	Papers	Number of papers about neurpathic pain/total number of papers	Citations (WOS)	Citations per paper	Open access	WOS sort	IF (2020)	Quartile	H-index
Diabetes	65	65/1422	241	3.71	8	ENDOCRINOLOGY & METABOLISM	9.461	Q1	7
Diabetologia	59	59/1422	75	1.27	3	ENDOCRINOLOGY & METABOLISM	10.122	Q1	2
Diabetic medicine	42	7/237	295	7.02	4	CLINICAL NEUROLOGY; NEUROSCIENCES	4.359	Q3	7
Journal of the peripheral nervous system	39	13/474	13	0.33	0	ENDOCRINOLOGY & METABOLISM	3.494	Q3	3
Value in health	32	32/1422	8	0.25	27	ECONOMICS; HEALTH CARE SCIENCES & SERVICES; HEALTH POLICY & SERVICES	5.728	Q2	1
Pain	31	31/1422	1638	52.84	10	CLINICAL NEUROLOGY; NEUROSCIENCES	6.961	Q1; Q2	19
Journal of pain	30	5/237	290	9.67	10	ANESTHESIOLOGY; CLINICAL NEUROLOGY; NEUROSCIENCES	5.828	Q2	6
Neurology	26	13/711	399	15.35	4	CLINICAL NEUROLOGY	9.91	Q1	4
Pain medicine	26	13/711	427	16.42	22	ANESTHESIOLOGY; MEDICINE, GENERAL & INTERNAL	3.75	Q3	13
Diabetes care	17	17/1422	1131	66.53	16	ENDOCRINOLOGY & METABOLISM	19.112	Q1	13

**FIGURE 4:**
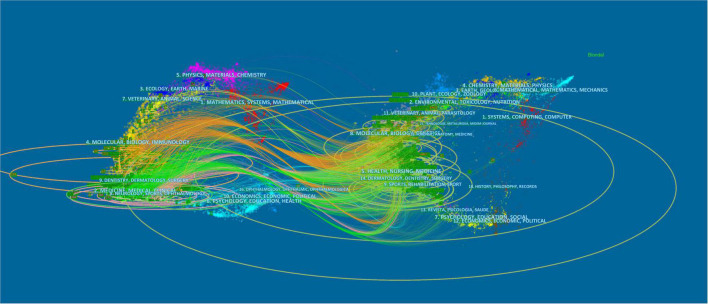
The dual-diagram overlay of journals related to DPNP research.

Figure 4 depicted the dual diagram of citing and cited of different journals, with the diagram on the left and right sides representing the citing journals and cited journals, respectively. The line between the citing and cited journals represented communication and connection between the two and the node tags meant the disciplines wrapped by the different journals. The ellipse’s aclinic axis represented the numbers of relevant authors, while the perpendicular axis was the amounts of journals published. Based on the diagram, the journals that contributed the most were mainly from the fields of *mathematic, medicine*, and *ecology*, while the journals cited the most were mainly from the fields of *systems*, *environmental*, and *earth*.

### Distribution by Different Countries and Institutions

**FIGURE 5:**
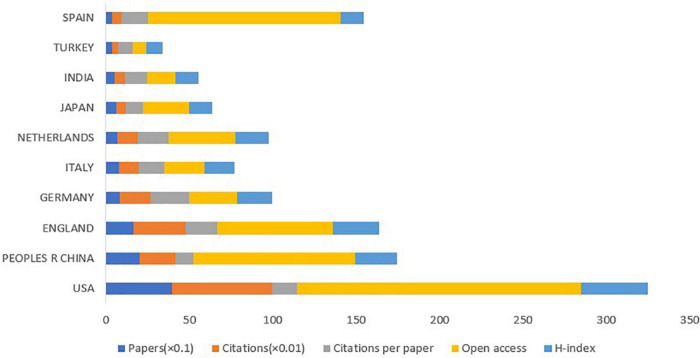
The amounts of papers, citations, citations per paper, open access papers, and H-index of the top 10 countries.

**FIGURE 6:**
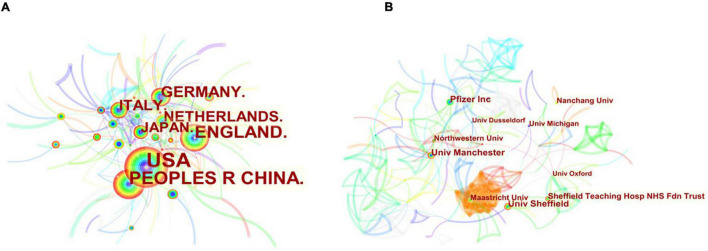
The analysis of countries and institutions. **(A)** Network diagram of countries worked on DPNP research. **(B)** Network diagram of institutions worked on DPNP research.

**FIGURE 7:**
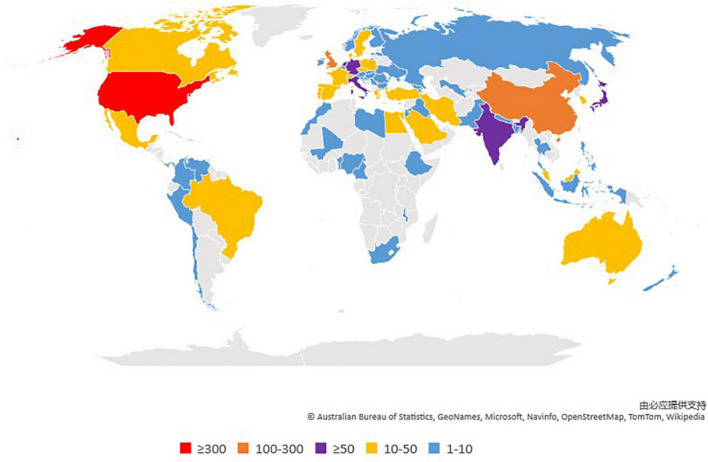
World diagram of total country output based on DPNP.

The Figure 5 showed the top 10 countries in terms of the numbers of publications on DPNP, with total 1,166 papers. The highest amounts of publications, citations, open access value, and the H-index was reported in United States, being 394, 6,003, 170, and 40, respectively; followed by China (200 publications); England (165 publications); and Germany (80 publications). Figure 6A showed the cooperation among various countries. The Czech Republic had the maximum centrality (0.50), which was followed by Switzerland (0.35) and India (0.33). According to the relevant definition of centrality, these countries indicated close cooperative links with other countries and represented some academic influence. Combining publication and centrality analysis, United States and China were in the dominant positions. The United States, England, Japan, and China have established extensive cooperative relationship and radiate outwards. A world diagram was created with the amounts of published papers to provide a clearer picture of the 1,422 published papers in each country (Figure 7). In this map, the Americas was in the ascendancy, with the United States leading the way.

**FIGURE 8:**
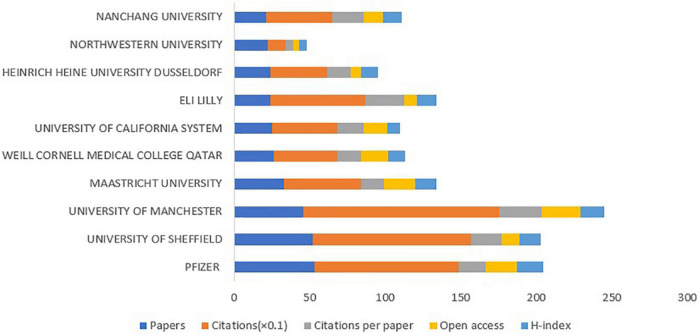
The amounts of papers, citations, citations per paper, open access papers, and H-index of the top 10 institutions.

The top 10 institutions by amounts of published papers were shown in Figure 8, which contributed 326 papers in the domain of DPNP. The Pfizer had the largest publications with the amount of 53. Although the Pfizer had the highest H-index value of 17, the University of Manchester had the most citations, citations per paper, and open access at 1,291, 28.07, and 26, respectively. Figure 6B depicted the cooperation and communication among various institutions. The Aarhus University showed maximum centrality (0.18), followed by University of Michigan (0.17) and Weill Cornell Medical College Qatar (0.15). These institutions exhibited extensive cooperative relationships and strong academic influence. Based on the analysis of the number and centrality of publications, Pfizer, Northwestern University and University of Manchester showed relatively close cooperative relationship.

### Distribution by Different Authors

**FIGURE 9:**
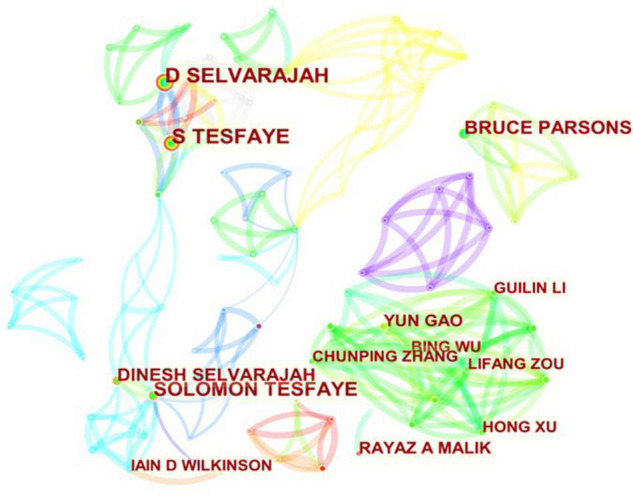
The analysis of authors. Network diagram of influential authors devoted to DPNP research.

The top 10 authors, co-cited authors, and co-cited references on DPNP research were listed in Table 2. Among the top 10 authors, Tesfaye S had the most publications (63 publications), followed by Selvarajah D (53 publications), and Wilkinson ID (29 publications). From perspective of co-cited authors, Tesfaye S also had the most cited times (271 cited times), followed by Ziegler D (166 cited times), and Boulton AJM (160 cited times). The collaboration among different authors was demonstrated in Figure 9.

**TABLE 2:** The top 10 authors, co-cited authors, and co-cited references on DPNP research.

Author	Published articles	Cocited author	Cited times	Cocited reference	Cited times
TESFAYE S	63	TESFAYE S	271	Painful diabetic peripheral neuropathy: consensus recommendations on diagnosis, assessment and management?	181
SELVARAJAH D	53	ZIEGLER D	166	The Pain in Neuropathy Study (PiNS): a cross-sectional observational study determining the somatosensory phenotype of painful and painless diabetic neuropathy?	133
WILKINSON ID	29	BOULTON AJM	160	A new look at painful diabetic neuropathy	49
PARSONS B	27	ABBOTT CA	143	Healthcare utilization and costs in diabetes relative to the clinical spectrum of painful diabetic peripheral neuropathy	56
MALIK RA	25	DWORKIN RH	137	Prevalence and characteristics of painful diabetic neuropathy in a large community-based diabetic population in the united kingdom	411
GANDHI R	21	BRIL V	135	Microvascular perfusion abnormalities of the thalamus in painful but not painless diabetic polyneuropathy a clue to the pathogenesis of pain in type 1 diabetes	47
GAO Y	20	DAVIES M	130	LncRNA NON-RATT021972 siRNA regulates neuropathic pain behaviors in type 2 diabetic rats through the P2X (7) receptor in dorsal root ganglia	74
ZIEGLER D	20	FINNERUP NB	119	From guideline to patient: a review of recent recommendations for pharmacotherapy of painful diabetic neuropathy?	61
FABER CG	16	GORE M	118	Spinal cord stimulation and pain relief in Painful diabetic peripheral neuropathy: a prospective two-center randomized controlled trial?	82
GREIG M	15	VINIK AI	110	Painful and painless diabetic neuropathies: what is the difference?	36

### Analysis by Different References

**FIGURE 10:**
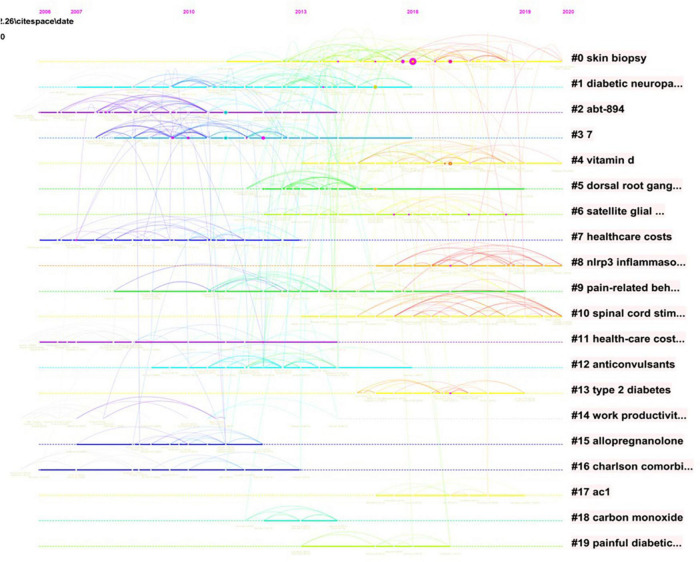
The analysis of references. Co-citation diagram (timeline view) of references from publications on DPNP research.

Figure 10 showed the time plot of the first 16 co-citation references in the cluster analysis. The modularity Q value was 0.8304 (higher than 0.5), which indicated the network was compatibly distributed to loosely coupled clusters. The largest cluster #0 was “*skin biopsy*”, followed by “*diabetic neuropathic pain*” (#1), “*abt-894*” (#2), and “*7*” (#3) (Chen and Wang, 2020).

### Analysis by Different Keywords

**FIGURE 11:**
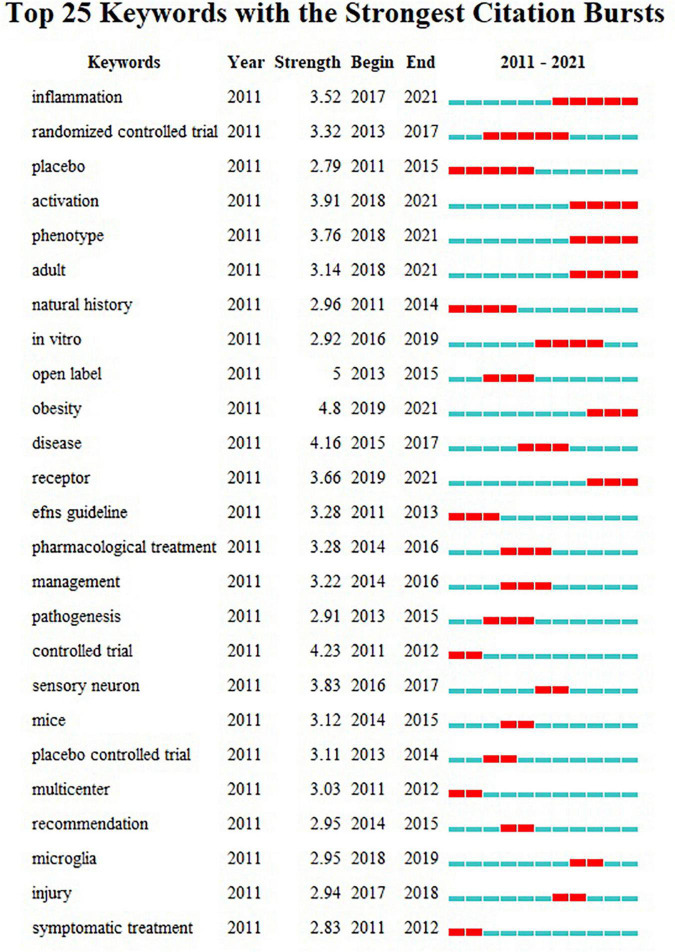
The keywords with the strongest citation bursts of publications on DPNP research.

The top 25 keywords with the strongest citation bursts from 2011 to 2021 was depicted in Figure 11. The strongest citation bursts of keyword since 2011 was *symptomatic treatment*. By the end of 2021, the keywords with the most outbreaks of cited literature included “*inflammation*” (2017–2021), “*activation*” (2018–2021), “*phenotype*” (2018–2021), “*phenotype*” (2018–2021), “*adult*” (2018–2021), and “*receptor*” (2019–2021) among the top 25 keywords (*inflammation, randomized controlled trial, placebo, activation, phenotype, adult, natural history, in vitro, open label, obesity, disease, receptor, EFNS guideline, pharmacological treatment, management, pathogenesis, controlled trial, sensory neuron, mice, placebo controlled trial, multicenter, recommendation, microglia, injury, and symptomatic treatment*).

### Analysis by the 10 Most Frequently Cited Papers

The top 10 frequently cited papers on DPNP were listed in Table 3. The most cited paper (411 citations) by Abbott with the title “*Prevalence and Characteristics of Painful Diabetic Neuropathy in a Large Community-Based Diabetic Population in the United Kingdom*” (Abbott et al., 2011) was published in 2011 in *Diabetes care*. Out of the top 10 citations, three of them were published in the journals with IF ≥ 10 (*Neuron* and *Diabetes care*) (Abbott et al., 2011; Tesfaye et al., 2013a; Feldman et al., 2017), four in journals with 5 ≤ IF < 10 (*Neurology* and *Pain*) (Bril et al., 2011; Yarnitsky et al., 2012; Tesfaye et al., 2013b; Themistocleous et al., 2016), one in journal with 3 ≤ IF < 5 (*Diabetes-Metabolism research and reviews*) (Tesfaye et al., 2011), and two in journals with 2 ≤ IF < 3 (*Current Medical research and opinion* and *Fitoterapia*) (Schwartz et al., 2011; Kandhare et al., 2012).

**TABLE 3:** The top 10 papers with the most citations on DPNP research.

Title	First author	Journal	IF (2019)	Year	Citations (WOS)	WOS sort	Category ranking
Prevalence and characteristics of painful diabetic neuropathy in a large community-based diabetic population in the United Kingdom	Abbott, CA	DIABETES CARE	19.112	2011	411	Endocrinology & metabolism	4/143
Conditioned pain modulation predicts duloxetine efficacy in painful diabetic neuropathy	Yarnitsky, D	PAIN	6.961	2012	320	Anesthesiology; Clinical Neurology; Neurosciences	6/32;25/204;43/272
Evidence-based guideline: Treatment of painful diabetic neuropathy	Bril, V	NEUROLOGY	9.91	2011	289	Clinical Neurology	10/204
New Horizons in diabetic neuropathy: mechanisms, bioenergetics, and pain	Feldman, EL	NEURON	17.173	2017	288	Neurosciences	6/272
Painful diabetic peripheral neuropathy: consensus recommendations on diagnosis, assessment and management	Tesfaye, S	DIABETES-METABOLISM RESEARCH AND REVIEWS	4.876	2011	181	Endocrinology & Metabolism	64/143
Mechanisms and management of diabetic painful distal symmetrical polyneuropathy	Tesfaye, S	DIABETES CARE	19.112	2013	170	Endocrinology & Metabolism	4/143
Safety and efficacy of tapentadol ER in patients with painful diabetic peripheral neuropathy: results of a randomized-withdrawal, placebo-controlled trial	Schwartz, S	CURRENT MEDICAL RESEARCH AND OPINION	2.58	2011	168	Medicine, General & Internal; Medicine, Research & Experimental	61/165;93/139
Duloxetine and pregabalin: High-dose monotherapy or their combination? The “COMBO-DN study” - a multinational, randomized, double-blind, parallel-group study in patients with diabetic peripheral neuropathic pain	Tesfaye, S	PAIN	6.961	2013	145	Anesthesiology; Clinical Neurology; Neurosciences	6/32;25/204;43/272
Neuroprotective effect of naringin by modulation of endogenous biomarkers in streptozotocin induced painful diabetic neuropathys	Kandhare, AD	FITOTERAPIA	2. 882	2012	136	Chemistry, Medicinal; Pharmacology & Pharmacy	38/61;152/271
The pain in neuropathy study (PiNS): a cross-sectional observational study determining the somatosensory phenotype of painful and painless diabetic neuropathy	Themistocleous, AC	PAIN	6.961	2016	133	Anesthesiology; Clinical Neurology; Neurosciences	6/32;25/204;43/272

## Discussion

### Global Development Tendency of Diabetic Peripheral Neuropathic Pain Research

This bibliometric analysis provided a scientific review of DPNP over the past 11 years (Chen and Wang, 2020). The numbers of publications correlated with DPNP have showed a continuous but unstable growth trend yearly, with the most obvious growth trend from 2014 to 2015. However, the numbers of citations have been drawn from 2,893 in 2011 to 138 in 2021. Notably, the increased amounts of published articles do not mean the improvement of the literature quality, and the decreased amounts of citations are not equal to the decline of quality. Considering the factor of year, the amounts of citations in the last 2 years are not as high as those in previous years, which is a normal phenomenon (Wang et al., 2020). The highest amounts of published papers and open access values are in 2019–2020, which are 285 and 135, respectively. Moreover, the year 2011–2012 has the highest number of citations (5,080) and H-index (39). These results indicate that DPNP is receiving increasing attention, and the papers published between 2011 and 2013 are of high-quality.

With regards of journals, *Diabetes* (65 publications), *Diabetologia* (59 publications), and *Diabetic medicine* (42 publications) contributed the most to the numbers of published papers. Among the top 10 journals, 45.45% of them were Q1, 27.27% were Q2, and 27.27% were Q3. IFs of 70% of the journals were more than 5. These results indicated that the *Diabetes* was the most influential journal in the field. Most of the top 10 journals had high IFs though some of them located in Q2 and Q3, which might imply that more exploration is required in this domain.

According to the numbers of papers published in the field of DPNP, the United States ranked first (394 publications), far ahead of China (200 publications), England (165 publications), Germany (80 publications), and other countries. The six European countries, three Asian countries, one North American country composed the top 10 countries. Figure 6 depicted a wide range of cooperative relationships that have been established between various countries/regions and institutions. From the top 10 institutions, 80% were the world-renowned universities. These results demonstrated that universities were a major front in the field of DPNP, with some American and European universities having a relatively large influence in this field. Pfizer, Northwestern University, and University of Manchester have relatively close cooperative relationship, meaning that the University and the company have established a wide cooperative relationship, which can play a certain positive role in the transformation from scientific research into clinical practice.

### Research Focusing on Diabetic Peripheral Neuropathic Pain

According to the subject sort of DPNP, the research mainly focused on *Endocrinology Metabolism*, *Clinical Neurology*, and *Neurosciences*. The prevention and education of diabetes have been emphasized based on the concept of *Endocrinology and Metabolism* (Sanjari et al., 2021). However, the largest amounts of citations, open access papers, and H-index were found in *Neurosciences*, being 4,771, 96, and 36, respectively. In terms of co-cited references, the largest cluster #0 was “*skin biopsy*”, followed by “*diabetic neuropathic pain*” (#1), “*abt-894*” (#2), and “*7*” (#3). Based on the analysis of keywords, while the strongest citation bursts of keyword since 2011 was *symptomatic treatment*, the keywords by the end of 2021 included “*inflammation*” (2017–2021), “*activation*” (2018–2021), “*phenotype*” (2018–2021), “*adult*” (2018–2021), and “*receptor*” (2019–2021). This change may represent that the focuses of research about DPNP have transferred from the superficial symptoms to the possible pathogenesis, which probably indicates that future research should further explore the mechanism of diabetes.

### Benefits and Limitations

This paper is the first to present a visual analysis of global trends and feasibility in the domain of DPNP over the last 11 years. The included publications are from different academic journals in the SCI-Expanded of WOS to obtain richer data and draw more convincing conclusions (Chen and Wang, 2020). Furthermore, some high-quality journals, such as *Diabetes care* (IF = 19.112), *Diabetologia* (IF = 10.122), and *Neurology* (IF = 9.91), are also included in this study. Moreover, this study contains a more comprehensive analysis, including the number and growth trend of annual publications, different subject sort of WOS, relationship among different journals, authors, countries and institutions, analysis by different references, citations, and keywords.

However, this study still has a few limitations. First of all, we only collected the English publications from SCI-expanded, which might neglect high-quality literature in other languages. In addition, some literatures use specific indicators of bibliometrics, such as Price’s Law, Lotka’s Law, and Bradford’s Law (López-Muñoz et al., 2013, 2015; Redondo et al., 2017), which could accurately reflect the changes in this field. Finally, the figure illustrating the links among different countries and institutions is hard to read due to the close and complicated cooperation among various states and institutions in this field.

## Conclusion

The study extracted some hidden and useful information from the 2011 to 2021 study of DPNP. The tendency of publications each year increased from 103 in 2011 to 127 in 2021, showing a continuous but unstable growth trend. *Endocrinology Metabolism* and *Clinical Neurology* were the two most popular subject sorts in this domain. The United States has occupied a leading position in this field and has established contacts with Italy, Japan, and other countries. The Pfizer had the largest publications with the amounts of 53. Tesfaye S published the most amounts of studies. The recent emerging burst keywords included “*inflammation*,” “*activation*,” “*phenotype*,” “*adult*,” and “*receptor.*” The study provided some valuable information for follow-up researchers, such as development trend and direction, influential journals, and cooperation among different regional institutions.

## Data Availability Statement

The original contributions presented in the study are included in the article/Supplementary Material, further inquiries can be directed to the corresponding author/s.

## Author Contributions

S-HD performed the data analyses and wrote the manuscript. Y-LZ helped perform the analysis with constructive discussions. Y-HZ and M-WW helped further revise and rewrote the manuscript. X-QW contributed to the conception of the study. All authors contributed to the article and approved the submitted version.

## Conflict of Interest

The authors declare that the research was conducted in the absence of any commercial or financial relationships that could be construed as a potential conflict of interest.

## Publisher’s Note

All claims expressed in this article are solely those of the authors and do not necessarily represent those of their affiliated organizations, or those of the publisher, the editors and the reviewers. Any product that may be evaluated in this article, or claim that may be made by its manufacturer, is not guaranteed or endorsed by the publisher.
