# Supplementary material for: The Last Decade Publications on Diabetic Peripheral Neuropathic Pain: A Bibliometric Analysis
Source: Front Mol Neurosci. 2022 Apr 13;15:854000. doi: 10.3389/fnmol.2022.854000 (PMC9043347; doi:10.3389/fnmol.2022.854000)
Supplement: Supplementary file 1 [file Table_1.DOCX]

**Supplementary1** Search strategy for Web of Science

#1 TI= (pain or pains or painful or neuralgia)

#2 TI= (diabete or diabetic or diabetes OR diabetics)

#3 #1 AND #2

Timespan=2011.01.01-2021.12.26 Databases=SCI-EXPANDED. LANGUAGE: (English)
